# Crystal structure of 1,2-bis­[(2-*tert*-butyl­phen­yl)imino]­ethane

**DOI:** 10.1107/S2056989015008610

**Published:** 2015-05-09

**Authors:** Alexandre C. Silvino, Juliana M. Torres

**Affiliations:** a Instituto de Macromoléculas Professora Eloisa Mano, Universidade Federal do Rio de Janeiro, CT, Bloco J, Ilha do Fundão, Rio de Janeiro, RJ 21945-970, Brazil

**Keywords:** crystal structure, di­imine, non-classical hydrogen bonds, DNA

## Abstract

The whole molecule of the title compound, C_22_H_28_N_2_, (I), is generated by inversion symmetry. The mol­ecule is rather similar to that of 2,3-bis­[(2-*tert*-butyl­phen­yl)imino]­butane, (II), a di­imine ligand comprising similar structural features [Ferreira *et al.* (2006 ▸). *Acta Cryst.* E**62**, o4282–o4284]. Both ligands crystallize with the –N=C(*R*)—C(*R*)=N– group around an inversion centre, in a *trans* configuration. Comparing the two structures, it may be noted that the independent planar groups in both mol­ecules [the central link, –N=C(*R*)—C(*R*)=N–, and the terminal aromatic ring] subtend an angle of 69.6 (1)° in (II) and 49.4 (2)° in (I). Ferreira and co-workers proposed that such angle deviation may be ascribed to the presence of two non-classical intra­molecular hydrogen bonds and steric factors. In fact, in (I), similar non-classical hydrogen bonds are observed, and the larger angular deviation in (II) may be assigned to the presence of methyl groups in the di­imino fragment, which can cause steric hindrance due to the presence of bulky *tert*-butyl substituents in the aromatic rings. The C=N bond lengths are similar in both compounds and agree with comonly accepted values.

## Related literature   

For general properties of di­imines, see: Rix & Brookhart (1995 ▸); Hissler *et al.* (2000 ▸); Ramakrishnan *et al.* (2011*a* ▸). For the inter­action of di­imine–metal complexes with DNA, see: Wang *et al.* (2004 ▸); Tan *et al.* (2008 ▸); Ramakrishnan *et al.* (2011*b* ▸). For a related structure, see: Ferreira *et al.* (2006 ▸).
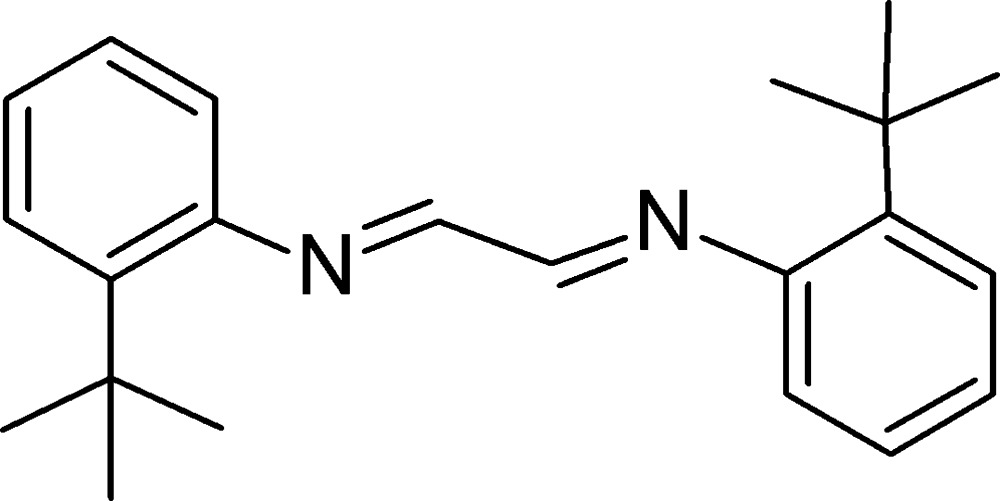


## Experimental   

### Crystal data  

C_22_H_28_N_2_
*M* *_r_* = 320.46
Monoclinic, 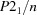

*a* = 12.333 (3) Å
*b* = 6.4740 (13) Å
*c* = 12.519 (3) Å
β = 95.22 (3)°
*V* = 995.5 (3) Å^3^
*Z* = 2
Mo *K*α radiation
μ = 0.06 mm^−1^
*T* = 293 K
0.3 × 0.17 × 0.07 mm

### Data collection  

Nonius KappaCCD diffractometer
21498 measured reflections
1811 independent reflections
1284 reflections with *I* > 2σ(*I*)
*R*_int_ = 0.072

### Refinement  

*R*[*F* ^2^ > 2σ(*F* ^2^)] = 0.049
*wR*(*F* ^2^) = 0.119
*S* = 1.06
1811 reflections
109 parameters
H-atom parameters constrained
Δρ_max_ = 0.13 e Å^−3^
Δρ_min_ = −0.15 e Å^−3^

### 

Data collection: *COLLECT* (Nonius, 2004 ▸); cell refinement: *DIRAX/LSQ* (Duisenberg, 1992 ▸); data reduction: *EVALCCD* (Duisenberg *et al.*, 2003 ▸); program(s) used to solve structure: *SHELXS97* (Sheldrick, 2008 ▸); program(s) used to refine structure: *SHELXL97* (Sheldrick, 2008 ▸); molecular graphics: *ORTEP-3 for Windows* (Farrugia, 2012 ▸); software used to prepare material for publication: *WinGX* (Farrugia, 2012 ▸).

## Supplementary Material

Crystal structure: contains datablock(s) global, I. DOI: 10.1107/S2056989015008610/bg2548sup1.cif

Structure factors: contains datablock(s) I. DOI: 10.1107/S2056989015008610/bg2548Isup2.hkl

**Fig. 1.**
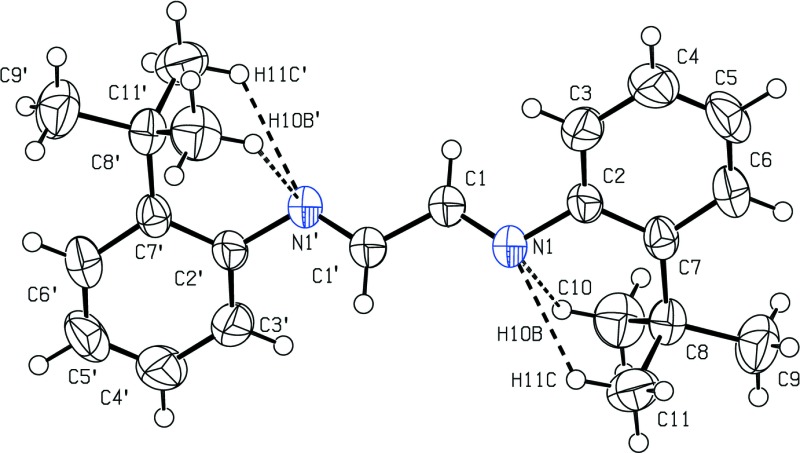
View of (I) (50% probability displacement ellipsoids). The dashed lines indicate the proposed non-classical intramolecular hydrogen bonds. [Symmetry code: (') 1 - x, 1 - y, -z.]

**Fig. 2.**
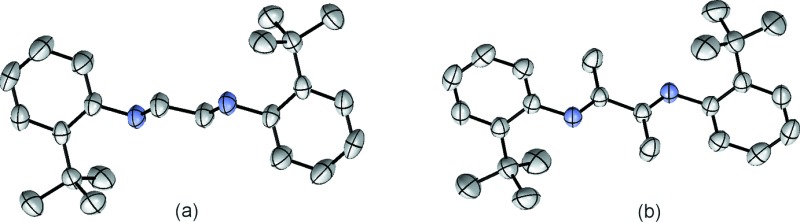
Comparison of the structures of (a) (I) and (b) (II).

Supporting information file. DOI: 10.1107/S2056989015008610/bg2548Isup3.cml

x y z . DOI: 10.1107/S2056989015008610/bg2548fig1.tif
View of (I) (50% probability displacement ellipsoids). The dashed lines indicate the proposed non-classical intra­molecular hydrogen bonds. [Symmetry code: (’) 1 − *x*, 1 − *y*, −*z*.]

a b . DOI: 10.1107/S2056989015008610/bg2548fig2.tif
Comparison of the structures of (*a*) (I) and (*b*) (II).

CCDC reference: 1062877

Additional supporting information:  crystallographic information; 3D view; checkCIF report

## Figures and Tables

**Table 1:** Hydrogen-bond geometry (, ) -

DHA	DH	HA	DA	DHA
C10H10*B*N1	0.960	2.405(2)	3.055(3)	124.8(3)
C11H11*C*N1	0.960	2.414(2)	3.064(3)	124.6(2)

## supplementary crystallographic information

### S1. Chemical context

The design and development of effective anti­cancer metallodrugs has become one of the more important areas in pharmaceutical industry and academia. Nonetheless, side effects associated with these complexes and the development of tumor resistance has led to the search for new generations of metal based anti­cancer agents. Di­imine compounds have been employed in many applications, including olefin polymerization, luminescence studies and metallodrug synthesis. (Rix & Brookhart, 1995; Hissler *et al.*, 2000; Ramakrishnan *et al.*, 2011a) The inter­action of di­imine Cobalt, Ruthenium and Iron complexes with DNA has attracted much attention during the last decade. (Wang *et al.*, 2004; Tan *et al.* 2008; Ramakrishnan *et al.*, 2011b). The anti­tumoral screening activity of potential metallodrugs with distinct nitro­gen based ligands has helped researchers to understand how factors as size, geometry and electronic structure can contribute to DNA binding thus allowing to categorize which factors are important to enhance metallodrug performance. We report herein on the crystal structure of C_22_H_28_N_2_ (I)

### S2. Structural commentary

The crystal structure of 2,3-Bis(2-tert-butyl­phenyl­imino)­butane, C_24_H_32_N_2_ (II), a di­imine ligand comprising similar structural features was already reported. (Ferreira *et al.*, 2006). Both ligands crystallize with the –N=C(R)—C(R)=N– group around an inversion centre, in a trans conﬁguration. Comparing the two structures, it may be noted that the independent planar groups in both molecules (the central link, –N=C(R)—C(R)=N–, and the terminal aromatic ring) subtend an angle of 69.6 (1)° in (II) and 49.4 (2)° in (I). Ferreira and co-workers proposed that such angle deviation may be ascribed to the presence of two non classical hydrogen bonds and steric factors. In fact, in the title compound, similar non-classical hydrogen bonds were observed: C10—H10B···N1 and C11—H11C···N1. (Fig 1 and Table 1) The greater angle deviation in (II) may be assigned to the presence of methyl groups in the di­imino fragment, which can cause steric hindrance due to the presence of bulky tert-butyl substituents in the aromatic rings. The C=N bond lengths are similar to the corresponding ones in (II) and agree well with what is expected for this bonding mode.

### S3. Synthesis and crystallization

To a solution of 2-tert-butyl­aniline (3.6 g; 26 mmol) in 15 mL de methanol, 1.5 mL of glyoxal solution (40 % in water; 13 mmol) was added. The resulting mixture was stirred overnight at room temperature. The yellow precipitate was filtered off, dried under vacuum for 2 days. Slow evaporation of the filtrate gave crystals suitable for single-crystal XRD studies. (Yield: 90 %)

### S4. Refinement

Crystal data, data collection and structure refinement details are summarized in Table 1. The H atoms were placed into the calculated idealized positions. All H atoms were refined with fixed individual displacement parameters [*U*_iso_(H) = 1.2U_eq_ (Csp^2^) or 1.5U_eq_ (methyl group)] using a riding model.

### Crystal data

**Table:**

C_22_H_28_N_2_	*F*(000) = 348
*M**_r_* = 320.46	*D*_x_ = 1.069 Mg m^−^^3^
Monoclinic, *P*2_1_/*n*	Mo *K*α radiation, λ = 0.71073 Å
Hall symbol: -P 2yn	Cell parameters from 21498 reflections
*a* = 12.333 (3) Å	θ = 3.6–25.4°
*b* = 6.4740 (13) Å	µ = 0.06 mm^−^^1^
*c* = 12.519 (3) Å	*T* = 293 K
β = 95.22 (3)°	Plate, yellow
*V* = 995.5 (3) Å^3^	0.3 × 0.17 × 0.07 mm
*Z* = 2	

### Data collection

**Table:**

Nonius KappaCCD diffractometer	1284 reflections with *I* > 2σ(*I*)
Horizonally mounted graphite crystal monochromator	*R*_int_ = 0.072
Detector resolution: 9 pixels mm^-1^	θ_max_ = 25.4°, θ_min_ = 3.6°
CCD scans	*h* = −14→14
21498 measured reflections	*k* = −7→7
1811 independent reflections	*l* = −15→15

### Refinement

**Table:**

Refinement on *F*^2^	0 restraints
Least-squares matrix: full	Hydrogen site location: inferred from neighbouring sites
*R*[*F*^2^ > 2σ(*F*^2^)] = 0.049	H-atom parameters constrained
*wR*(*F*^2^) = 0.119	*w* = 1/[σ^2^(*F*_o_^2^) + (0.0452*P*)^2^ + 0.2479*P*] where *P* = (*F*_o_^2^ + 2*F*_c_^2^)/3
*S* = 1.06	(Δ/σ)_max_ < 0.001
1811 reflections	Δρ_max_ = 0.13 e Å^−^^3^
109 parameters	Δρ_min_ = −0.15 e Å^−^^3^

### Special details

**Table:**

Geometry. All e.s.d.'s (except the e.s.d. in the dihedral angle between two l.s. planes) are estimated using the full covariance matrix. The cell e.s.d.'s are taken into account individually in the estimation of e.s.d.'s in distances, angles and torsion angles; correlations between e.s.d.'s in cell parameters are only used when they are defined by crystal symmetry. An approximate (isotropic) treatment of cell e.s.d.'s is used for estimating e.s.d.'s involving l.s. planes.

### Fractional atomic coordinates and isotropic or equivalent isotropic displacement parameters (Å^2^)

**Table:**

	*x*	*y*	*z*	*U*_iso_*/*U*_eq_	
N1	0.46712 (12)	0.1526 (2)	0.10801 (11)	0.0485 (4)	
C1	0.47499 (15)	0.0981 (3)	0.01174 (14)	0.0520 (5)	
H1	0.4486	0.1842	−0.0442	0.062*	
C2	0.41192 (14)	0.3400 (3)	0.12810 (12)	0.0440 (4)	
C3	0.31398 (16)	0.3836 (3)	0.06876 (15)	0.0622 (6)	
H3	0.2873	0.292	0.0155	0.075*	
C4	0.25541 (18)	0.5595 (4)	0.08691 (17)	0.0736 (7)	
H4	0.19	0.587	0.0465	0.088*	
C5	0.29551 (17)	0.6930 (3)	0.16573 (18)	0.0694 (6)	
H5	0.2576	0.8134	0.1783	0.083*	
C6	0.39173 (16)	0.6496 (3)	0.22646 (15)	0.0559 (5)	
H6	0.4171	0.7428	0.2795	0.067*	
C7	0.45274 (13)	0.4721 (2)	0.21188 (13)	0.0413 (4)	
C8	0.55778 (14)	0.4249 (3)	0.28333 (14)	0.0476 (4)	
C9	0.58580 (19)	0.5962 (4)	0.36621 (18)	0.0777 (7)	
H9A	0.5955	0.7244	0.3298	0.117*	
H9B	0.6518	0.5612	0.409	0.117*	
H9C	0.5276	0.6101	0.4117	0.117*	
C10	0.65538 (15)	0.4063 (4)	0.21568 (17)	0.0701 (6)	
H10A	0.6639	0.533	0.1775	0.105*	
H10B	0.6429	0.295	0.1654	0.105*	
H10C	0.7203	0.3791	0.2619	0.105*	
C11	0.54410 (18)	0.2230 (3)	0.34482 (16)	0.0684 (6)	
H11A	0.4834	0.2354	0.3872	0.103*	
H11B	0.6091	0.1959	0.3909	0.103*	
H11C	0.5313	0.1113	0.2948	0.103*	

### Atomic displacement parameters (Å^2^)

**Table:**

	*U*^11^	*U*^22^	*U*^33^	*U*^12^	*U*^13^	*U*^23^
N1	0.0604 (9)	0.0429 (9)	0.0420 (8)	0.0096 (7)	0.0034 (7)	−0.0089 (7)
C1	0.0685 (12)	0.0456 (10)	0.0416 (10)	0.0134 (9)	0.0028 (8)	−0.0051 (8)
C2	0.0546 (10)	0.0405 (10)	0.0380 (9)	0.0103 (8)	0.0092 (7)	−0.0021 (7)
C3	0.0662 (13)	0.0709 (14)	0.0482 (11)	0.0188 (11)	−0.0016 (9)	−0.0108 (10)
C4	0.0681 (14)	0.0872 (17)	0.0650 (13)	0.0346 (13)	0.0029 (11)	0.0000 (12)
C5	0.0734 (14)	0.0550 (13)	0.0827 (15)	0.0288 (11)	0.0235 (12)	0.0000 (11)
C6	0.0639 (12)	0.0415 (11)	0.0650 (12)	0.0040 (9)	0.0201 (10)	−0.0096 (9)
C7	0.0496 (10)	0.0348 (9)	0.0417 (9)	−0.0007 (8)	0.0171 (7)	−0.0024 (7)
C8	0.0514 (10)	0.0432 (10)	0.0489 (10)	−0.0055 (8)	0.0089 (8)	−0.0081 (8)
C9	0.0799 (15)	0.0730 (15)	0.0787 (15)	−0.0088 (12)	−0.0013 (12)	−0.0280 (12)
C10	0.0513 (11)	0.0795 (16)	0.0811 (15)	0.0017 (11)	0.0144 (10)	−0.0068 (12)
C11	0.0809 (14)	0.0659 (14)	0.0554 (12)	−0.0091 (12)	−0.0105 (10)	0.0094 (10)

### Geometric parameters (Å, º)

**Table:**

N1—C1	1.268 (2)	C7—C8	1.536 (2)
N1—C2	1.425 (2)	C8—C9	1.537 (2)
C1—H1	0.93	C9—H9A	0.96
C1—C1^i^	1.454 (3)	C9—H9B	0.96
C2—C3	1.388 (2)	C9—H9C	0.96
C2—C7	1.410 (2)	C8—C10	1.538 (2)
C3—C4	1.378 (3)	C10—H10A	0.96
C3—H3	0.93	C10—H10B	0.96
C4—C5	1.370 (3)	C10—H10C	0.96
C5—H5	0.93	C8—C11	1.534 (3)
C4—H4	0.93	C11—H11A	0.96
C5—C6	1.379 (3)	C11—H11B	0.96
C6—C7	1.395 (2)	C11—H11C	0.96
C6—H6	0.93		
			
C1—N1—C2	118.93 (15)	C11—C8—C9	107.72 (16)
N1—C1—C1^i^	120.4 (2)	C7—C8—C9	112.06 (15)
N1—C1—H1	119.8	C11—C8—C10	109.68 (16)
C1^i^—C1—H1	119.8	C7—C8—C10	110.83 (14)
C3—C2—C7	120.59 (16)	C9—C8—C10	106.81 (16)
C3—C2—N1	119.03 (15)	C8—C9—H9A	109.5
C7—C2—N1	120.24 (15)	C8—C9—H9B	109.5
C4—C3—C2	121.56 (19)	H9A—C9—H9B	109.5
C4—C3—H3	119.2	C8—C9—H9C	109.5
C2—C3—H3	119.2	H9A—C9—H9C	109.5
C5—C4—C3	118.67 (19)	H9B—C9—H9C	109.5
C5—C4—H4	120.7	C8—C10—H10A	109.5
C3—C4—H4	120.7	C8—C10—H10B	109.5
C4—C5—C6	120.33 (19)	H10A—C10—H10B	109.5
C4—C5—H5	119.8	C8—C10—H10C	109.5
C6—C5—H5	119.8	H10A—C10—H10C	109.5
C5—C6—C7	122.91 (18)	H10B—C10—H10C	109.5
C5—C6—H6	118.5	C8—C11—H11A	109.5
C7—C6—H6	118.5	C8—C11—H11B	109.5
C6—C7—C2	115.89 (16)	H11A—C11—H11B	109.5
C6—C7—C8	121.60 (15)	C8—C11—H11C	109.5
C2—C7—C8	122.51 (14)	H11A—C11—H11C	109.5
C11—C8—C7	109.64 (14)	H11B—C11—H11C	109.5
			
C2—N1—C1—C1^i^	−176.3 (2)	C3—C2—C7—C6	−3.0 (2)
C1—N1—C2—C3	44.2 (2)	N1—C2—C7—C6	−178.76 (15)
C1—N1—C2—C7	−139.97 (18)	C3—C2—C7—C8	176.94 (17)
C7—C2—C3—C4	2.2 (3)	N1—C2—C7—C8	1.2 (2)
N1—C2—C3—C4	178.05 (18)	C6—C7—C8—C11	117.87 (18)
C2—C3—C4—C5	−0.1 (3)	C2—C7—C8—C11	−62.0 (2)
C3—C4—C5—C6	−1.0 (3)	C6—C7—C8—C9	−1.7 (2)
C4—C5—C6—C7	0.1 (3)	C2—C7—C8—C9	178.39 (16)
C5—C6—C7—C2	1.9 (3)	C6—C7—C8—C10	−120.91 (18)
C5—C6—C7—C8	−178.02 (17)	C2—C7—C8—C10	59.2 (2)

Symmetry code: (i) −*x*+1, −*y*, −*z*.

## References

[bb2] Duisenberg, A. J. M. (1992). *J. Appl. Cryst.* **25**, 92–96.

[bb3] Duisenberg, A. J. M., Kroon-Batenburg, L. M. J. & Schreurs, A. M. M. (2003). *J. Appl. Cryst.* **36**, 220–229.

[bb4] Farrugia, L. J. (2012). *J. Appl. Cryst.* **45**, 849–854.

[bb5] Ferreira, L. C., Filgueiras, C. A. L., Hörner, M., Visentin, L. do C. & Bordinhao, J. (2006). *Acta Cryst.* E**62**, o4282–o4284.

[bb6] Hissler, M., Connick, W. B., Geiger, D. K., McGarrah, J. E., Lipa, D., Lachicotte, R. J. & Eisenberg, R. (2000). *Inorg. Chem.* **39**, 447–457. 10.1021/ic991250n 11229561

[bb7] Nonius (2004). Nonius BV, Delft, The Netherlands.

[bb8] Ramakrishnan, S., Shakthipriya, D., Suresh, E., Periasamy, V. S., Akbarsha, M. A. & Palaniandavar, M. (2011*a*). *Inorg. Chem.* **50**, 6458–6471. 10.1021/ic1024185 21671566

[bb9] Ramakrishnan, S., Suresh, E., Akbarsha, M. A., Riyasden, A. & Palaniandavar, M. (2011*b*). *Dalton Trans.* **40**, 3524–3536. 10.1039/c0dt00466a 21369607

[bb10] Rix, F. C. & Brookhart, M. (1995). *J. Am. Chem. Soc.* **117**, 1137–1138.

[bb11] Sheldrick, G. M. (2008). *Acta Cryst.* A**64**, 112–122. 10.1107/S0108767307043930 18156677

[bb12] Tan, C., Liu, J., Chen, L., Shi, S. & Ji, L. (2008). *J. Inorg. Biochem.* **102**, 1644–1653. 10.1016/j.jinorgbio.2008.03.005 18468690

[bb13] Wang, X.-L., Chao, H., Li, H., Hong, X.-L., Liu, Y.-J., Tan, L.-F. & Ji, L. N. (2004). *J. Inorg. Biochem.* **98**, 1143–1150. 10.1016/j.jinorgbio.2004.04.003 15149826

