# Supplementary figures and images for: Crystal structure of 1,2-bis­[(2-tert-butyl­phen­yl)imino]­ethane
Source: Acta Crystallogr E Crystallogr Commun. 2015 May 9;71(Pt 6):o385–6. doi: 10.1107/S2056989015008610 (PMC4459346; doi:10.1107/S2056989015008610)

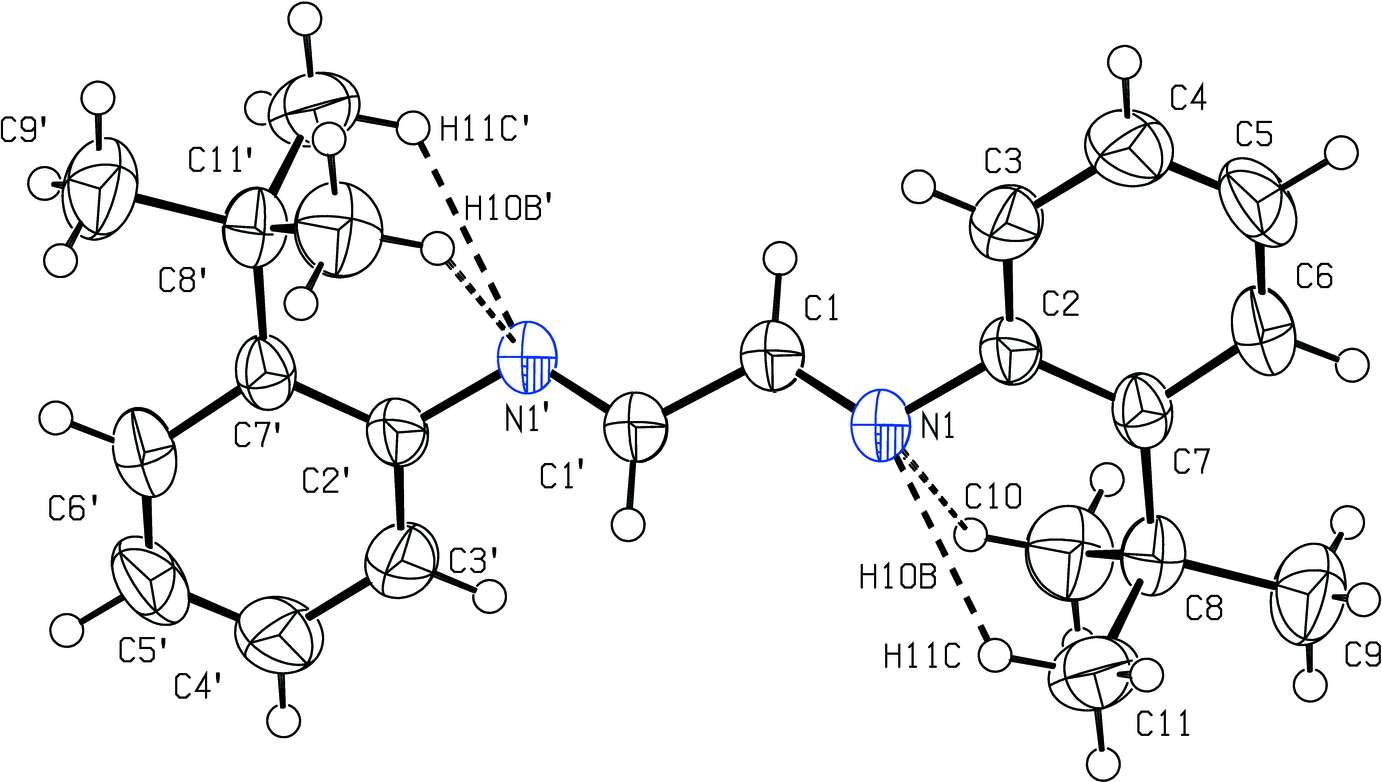

Supplement: Supplementary file 4 [file e-71-0o385-fig1.tif]

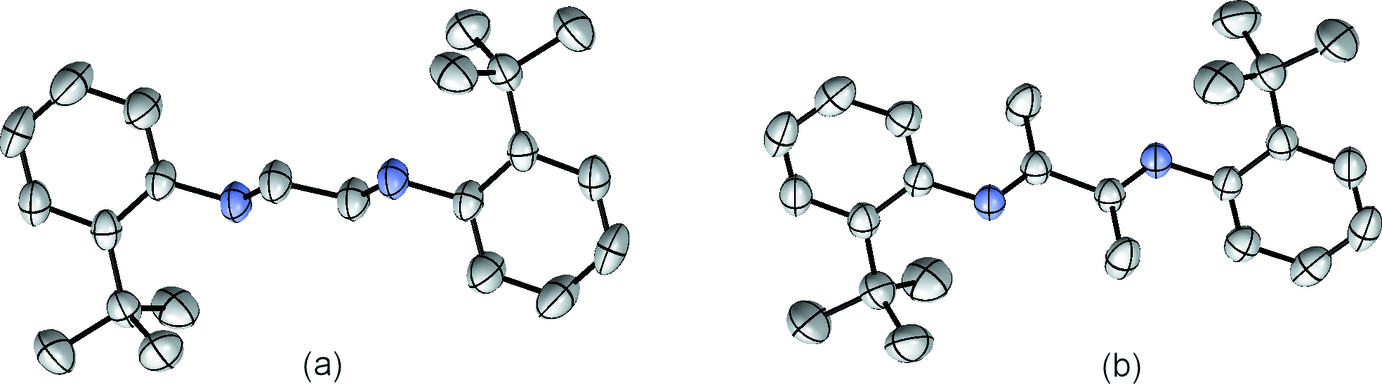

Supplement: Supplementary file 5 [file e-71-0o385-fig2.tif]
